# Heart Rate Variability in Insulo-Opercular Epilepsy

**DOI:** 10.3390/brainsci11111505

**Published:** 2021-11-13

**Authors:** Thi Phuoc Yen Tran, Philippe Pouliot, Elie Bou Assi, Pierre Rainville, Kenneth A. Myers, Manon Robert, Alain Bouthillier, Mark R. Keezer, Dang Khoa Nguyen

**Affiliations:** 1 CHUM Research Center, University of Montreal, Montreal, QC H2X 0A9, Canada; yen.tran@umontreal.ca (T.P.Y.T.); elie.bou.assi.chum@ssss.gouv.qc.ca (E.B.A.); manon.robert.chum@ssss.gouv.qc.ca (M.R.); m.keezer@outlook.com (M.R.K.); 2 Safe Engineering Services and Technologies, Laval, QC H7L 6E8, Canada; ph.pouliot@gmail.com; 3 Labeo Technologies, Montreal, QC H3V 1A2, Canada; 4 Department of Somatology, University of Montreal, Montreal, QC H3T 1J7, Canada; pierre.rainville@umontreal.ca; 5 Research Centre of Institut Universitaire de Gériatrie de Montréal, Montreal, QC H3C 3J7, Canada; 6 Research Institute of the McGill University Medical Centre, Montreal, QC H3H 2R9, Canada; kenneth.myers@mcgill.ca; 7 Division of Neurology, Department of Pediatrics, Montreal Children’s Hospital, McGill University Health Centre, Montreal, QC H4A 3J1, Canada; 8 Division of Neurosurgery, CHUM, University of Montreal, Montreal, QC H2X 0C1, Canada; alain.bouthillier@umontreal.ca; 9 Division of Neurology, CHUM, University of Montreal, Montreal, QC H2X 0C1, Canada

**Keywords:** heart rate variability, insulo-opercular epilepsy, cardiac autonomic dysfunction

## Abstract

Background: We aimed to evaluate heart rate variability (HRV) changes in insulo-opercular epilepsy (IOE) and after insulo-opercular surgery. Methods: We analyzed 5-min resting HRV of IOE patients before and after surgery. Patients’ SUDEP-7 risk inventory scores were also calculated. Results were compared with age- and sex-matched patients with temporal lobe epilepsy (TLE) and healthy individuals. Results: There were no differences in HRV measurements between IOE, TLE, and healthy control groups (and within each IOE group and TLE group) in preoperative and postoperative periods. In IOE patients, the SUDEP-7 score was positively correlated with pNN50 (percentage of successive RR intervals that differ by more than 50 ms) (*p* = 0.008) and RMSSD (root mean square of successive RR interval differences) (*p* = 0.019). We stratified IOE patients into those whose preoperative RMSSD values were below (Group 1a = 7) versus above (Group 1b = 9) a cut-off threshold of 31 ms (median value of a healthy population from a previous study). In group 1a, all HRV values significantly increased after surgery. In group 1b, time-domain parameters significantly decreased postoperatively. Conclusions: Our results suggest that in IOE, HRV may be either decreased in parasympathetic tone or increased globally in both sympathetic and parasympathetic tones. We found no evidence that insulo-opercular surgeries lead to major autonomic dysfunction when a good seizure outcome is reached. The increase in parasympathetic tone observed preoperatively may be of clinical concern, as it was positively correlated with the SUDEP-7 score.

## 1. Introduction

The role of the insula in drug-resistant epilepsy has been increasingly recognized with the report of several cases of insular, insulo-opercular or temporo-insular epilepsy successfully treated by resection, radio-frequency thermocoagulation, or laser ablation [1,2,3,4,5,6]. Because of the insula’s implication in autonomic regulation, which is supported by the insular electrical stimulation [7,8], a few studies have looked at possible evidence of autonomic dysfunction in insular epilepsy, with some authors even suggesting a higher risk of Sudden Unexpected Death in Epilepsy (SUDEP) [9,10].

Heart rate variability (HRV) is considered to be an index of autonomic activity that reflects brain–heart interactions and the balance between sympathetic and parasympathetic activity [11]. HRV has been shown to be a reliable biomarker of cardiovascular dysfunction in cardiovascular diseases [12,13,14,15,16] and is considered to be a promising early biomarker of cognitive impairment [17,18] and is increasingly used for the evaluation of autonomic changes in some neurological disorders, such as diabetic neuropathy [19], stroke [20], multiple sclerosis [21] and Parkinson’s disease [22].

Given that chronic autonomic dysfunction or cardiovascular dysfunction may be involved in the mechanisms of SUDEP [9,10], several studies since the late 1990s have measured HRV changes at different time points in the different populations of patients with epilepsy and have assessed its potential use as a biomarker for SUDEP. Notably, interictal HRV studies have been conducted on patients with newly diagnosed epilepsy [23,24,25], generalized epilepsy [26], Lennox-Gastaut syndrome [27], Dravet syndrome [28], frontal lobe epilepsy [29], and temporal lobe epilepsy (TLE) [30,31,32,33,34]. While most studies suggested the presence of resting HRV alterations in patients with refractory epilepsy [35], especially in patients with focal to bilateral tonic-clonic seizure [36], it is not clear whether HRV changes are associated with the risk of SUDEP [28,37,38,39,40,41]. Recently, an international, multicenter, retrospective and nested case-control study provided Class III evidence that, in patients with epilepsy, some measures of HRV are associated with SUDEP [42].

While several studies have looked at the effect of acute ischemic damage to the insula on HRV [43,44], the impact of chronic insular epilepsy, or insular epilepsy surgery on HRV, has not been studied thoroughly. In 2016, Lacuey et al. reported progressive changes in HRV prior to SUDEP in two patients with left insular damage from previous failed temporal/temporo-insular epilepsy surgeries [45]. One patient developed a progressive increase in parasympathetic tone and a decrease in sympathetic tone, whereas the other demonstrated opposite changes. The same group then examined HRV changes in 21 patients with epilepsy operated in the temporal lobe and a variable portion of the insula [46]. Authors found a significant decrease in root mean square difference of successive RR intervals (RMSSD) and coefficient of variation (CV) in patients who had <25% (*n* = 7) or ≥25% (*n* = 1) of their insula resected compared to those whose insula was not (*n* = 4) or minimally/marginally (*n* = 9) resected. Because most patients had temporal or temporal plus epilepsy, the effect of insular epilepsy per se remains unknown. Furthermore, because nearly half of patients continued to have seizures post-operatively, it is unclear whether HRV changes were due to insular surgery or persistent seizures. Finally, there was no control group to help account for the physiological decrease in HRV that occurs with aging.

In this study, we assessed pre- and post-operative HRV in patients with insulo-opercular epilepsy (IOE). Considering the role of the insula in autonomic and cardiovascular control, we hypothesized that HRV could be altered by long-standing insulo-opercular seizures, as well as insulo-opercular resections. We also sought to look at the relationship between HRV changes and SUDEP risk factors.

## 2. Materials and Methods

### 2.1. Study Population

We retrospectively reviewed all patients with IOE investigated and treated at our institution between 2000 and 2021. An insulo-opercular focus was assumed based on the presence of an epileptogenic lesion, a clear cluster of dipoles on magnetoencephalography, or intracranial EEG-recorded seizures in that area. Patients were included if: (1) they had at least one pre-operative video-electroencephalography (VEEG) monitoring study with electrocardiographic (ECG) lead derivations available for analysis review; and (2) had undergone epilepsy surgery in which insulo-opercular areas (with or without the ipsilateral temporal lobe) were part of the epileptogenic zone. Exclusion criteria were: (1) a history of cardiac disease (e.g., heart failure, cardiac ischemia, and arrhythmia) or any condition that could impair autonomic function (e.g., diabetes mellitus); and (2) use of antiarrhythmic agents (e.g., beta-blockers).

The study was performed in two parts. Since the primary purpose of our study was to determine HRV changes in IOE, in the first part of the study, we only included patients who became free or nearly free of disabling seizures for more than one year (Engel I or II) after epilepsy surgery limited to the insulo-opercular region (since a good postsurgical seizure outcome is the best way to make sure the seizure focus has been adequately identified). We excluded patients who had surgery outside the insulo-opercular area (in the same or in a previous surgery). For this first part of our study, an active control group and a healthy control group were established. The active control group consisted of 16 age- and sex-matched TLE patients who had a good seizure outcome after an anterior temporal lobectomy. Similar inclusion and exclusion criteria were used, except for the location of the seizure focus and surgery. Since for IOE and TLE patients, HRV comparisons were carried out twice, pre- and post-operatively, two separate healthy control groups were constituted, matched for age and sex with patients for both study periods.

In the second part of the study, because we wished to see whether HRV changes were different in patients with temporo-insular epilepsy compared to patients with IOE patients, and also whether failed insular surgery had a negative impact on HRV, we included all patients whose epilepsy surgery consisted of the removal of the insulo-opercular region with or without the ipsilateral temporal lobe and with or without a good seizure outcome. For HRV comparisons in the preoperative period, each patient was matched for sex and age with one healthy individual. For HRV comparisons in the postoperative period, an age-correction method (described in Section 2.5) was used to adjust HRV values of each healthy control at the postoperative age of the matched IOE patient.

Patients’ clinical charts were also carefully reviewed to retrieve demographic characteristics and clinical information, neuroimaging findings, scalp and intracranial VEEG results, details of the surgical treatment, and outcome. The revised SUDEP-7 risk inventory scores (SUDEP-7 score) were calculated from seven elements: (1) more than three tonic-clonic seizures in the last year; (2) one or more tonic-clonic seizures in the last year (if risk factor 1 selected, score as 0); (3) one or more seizures of any type over the last 12 months (if risk factor 4 selected, score as 0); (4) more than 50 seizures of any type per month over the last 12 months; (5) duration of epilepsy ≥ 30 years; (6) current use of three or more antiseizure medications; and (7) mental retardation, IQ < 70, or too impaired to test [38].

### 2.2. Recording Procedures

For each patient, 12 five-minute segments were selected from VEEG recordings performed during their presurgical assessment. Three lead ECGs were recorded and digitized at a sampling frequency of 200 Hz. Periods were selected based on the following criteria, inspired by Myers et al. [47]: (1) awake state during daytime (from 8 am to 4 pm); (2) fewer movement artifacts; (3) not during eating; (4) not during the hyperventilation procedure; (5) at least eight hours after the last focal to bilateral tonic-clonic seizure; (6) at least one hour after the last known clinical seizure (excluding focal to bilateral tonic-clonic seizures); and (7) at least one hour before the next clinical seizure. Since video recordings were not available during interictal periods (not archived), in order to best select the segment closest to resting state, the segment out of the 12 five-minute segments with the lowest mean heart rate was chosen for HRV analysis.

A one-hour continuous three-lead ECG awake recording at resting state in a lying position was carried out on all control subjects and epileptic patients in the post-operative periods. From that one-hour recording a 5-min segment was extracted for HRV analysis (generally from the 6th to the 10th minute unless it contained too many artifacts in which case the next 5-min segment was selected).

### 2.3. HRV Analysis

Consecutive R-R intervals were measured using the Brain Vision Analyser 2.1 commercial software (Brain Products GmbH, Gilching, Germany). R peaks were initially automatically detected from ECG data and then subsequently visually inspected to detect and manually correct artifacts, missed beats, or ectopic beats. Ectopic beats were removed from the recordings, and were replaced by an interpolated R-R interval [48]. The Kubios HRV 3.2.0 software (Kubios Oy, Kuopio, Finland) was used to calculate standard HRV parameters. Because the standard deviation of RR intervals (SDNN) is more accurate when calculated over 24 h rather than during short periods, only two time-domain parameters were analyzed: the root mean square of successive RR interval differences (RMSSD) and the percentage of successive RR intervals that differ by more than 50 ms (pNN50). Frequency-domain parameters, including low-frequency (LF) power (0.04–0.15 Hz) and high-frequency (HF) power (0.15–0.4 Hz), were obtained by applying the fast Fourier transform (FFT) to the RR interval tachogram (RR durations versus the number of progressive beats). We did not analyze the LF/HF ratio, as its role in reflecting the sympathovagal balances is controversial [48].

Out of the time-domain parameters, RMSSD is considered to be a primary measure to assess the vagal tone. Both RMSSD and pNN50 are mainly influenced by the parasympathetic nervous system. Out of the frequency-domain parameters, the HF power represents parasympathetic influences on the heart rate, while the LF power reflects predominantly sympathetic activity [48].

### 2.4. Statistical Analysis

The statistical analysis was performed using the Statistical package for the social sciences (SPSS) version 25 (IBM, New York, NY, USA).

Since data were not normally distributed and there was a small number of studied populations, non-parametric statistical tests were used. The median and interquartile ranges (IQR) were calculated for each HRV variable, and values were expressed as median (IQR). HRV values of all three groups (IOE, TLE, and control) were compared using the Kruskal–Wallis test. The Mann–Whitney U test was performed to compare two independent samples. Within a group, a Wilcoxon signed-rank test was used to compare pre-operative and post-operative HRV values. Spearman’s rank correlation coefficients were calculated to evaluate the relationship between HRV values and the SUDEP-7 score. The results were considered significant at *p* < 0.05.

### 2.5. Age Correction Methods

In part 2 of the study, pre-operative HRV changes of epileptic patients were compared to healthy controls matched for age and sex. Since the interval between time of surgery and post-operative HRV measures were different between epileptic patients, an age correction method was used to adjust HRV values of each healthy control at the postoperative age of the matched IOE patients, as previous studies have shown that both time- and frequency HRV features are age-dependent. In 2012, Voss et al. calculated reference values for 5-min resting HRV indices from a population of 1906 healthy subjects aged 25 to 74 years [42], which were fitted to quadratic regressions with a correlation coefficient (r) of more than 0.99 and a *p*-value < 0.01:

- For RMSSD: y = 75.78 − 1.618x + 0.011x^2^, r = 0.9982
- For pNN50: y = 60 − 1.89x + 0.015x^2^, r = 0.9989
- For LF: y = 310.114 − 6.6771x + 0.03857x^2^, r = 0.9964
- For HF: y = 271.214 − 7.4701x + 0.05407x^2^, r = 0.9983,

With y = HRV value, x = age.

The value determined for each parameter from this quadratic regression was considered as the “theoretical value.” The adjusted value of each parameter at the desired postoperative time point (age 2) was calculated by adding the theoretical value of each parameter at age 2 to the difference between the actual value and its theoretical value at age 1 (i.e., the actual age of the control individual in the preoperative period):

Adjusted value at age 2 = Theoretical value at age 2 + (actual value at age 1 − theoretical value at age 1).

This study was approved by our institutional ethics committee (Project 16.146). All participants signed written informed consent.

## 3. Results

### 3.1. Patients and Demographics

Sixteen patients (11 females) were enrolled in the first part of the study, aged from 19 to 47 years (median (IQR) = 34.50 (9)) at the time of presurgical evaluation. Findings from the presurgical evaluation, presurgical revised SUDEP-7 score, and surgical data for all patients are summarized in Table 1. Their SUDEP-7 score ranged from 1 to 6 (median (IQR) = 2 (2)). Details of the score components for each patient can be found in Appendix A Table A1. The epileptic focus was lateralized to the right side in 7 patients and the left in 9 patients. Median (IQR) epilepsy duration until pre-operative evaluation was 16.50 (15.50) years (range from 6 to 42 years). Duration from surgery to postoperative ECG recording ranged from 4 to 204 months (median (IQR) = 57.50(72)). Median (IQR) age at this post-operative period was 37.50 (16), ranging from 19 to 56 years. The surgical outcome was Engel I for 15 patients (13 Engel Ia and 2 Engel Ib) and Engel IId for one patient at the time of postoperative ECG recording.

The TLE group consisted of 11 females and 5 males, aged from 21–46 years (median (IQR) = 32.50 (12)) at the time of presurgical evaluation (Table 2). Median SUDEP-7 scores for TLE patients was 2 (3) (range 1–5), which was not significantly different to that of IOE patients. The seizure focus was lateralized to the right in 4 patients and to the left in 12. Epilepsy duration (IQR) for TLE patients was 15.5 (19.5) years. Duration from surgery to post-operative ECG recording ranged from 14 to 131 months (median (IQR) = 57 (50)). Median (IQR) age at the post-operative period was 38 (18) years (ranged from 24–54). The surgical outcome was Engel IA for all TLE patients.

Two control groups of 16 healthy subjects, matched for age and sex with the patients at the pre- and post-operative period, were established. Median age for pre-operative and postoperative control groups was 34 (10) and 37.5(16), respectively (Appendix A Table A2).

In the second part of the study, the group of interest was composed of twenty-seven patients (18 females) with IOE without or with temporal epilepsy. Preoperative age ranged from 19 to 54 (median age (IQR) = 35(10)). Postoperative age ranged from 19 to 56 (median (IQR) = 38 (15)). A summary of their demographic and clinical findings can be found in Table 3. The control group consisted of 27 subjects matched for sex and age with IOE patients at the preoperative period (Appendix A Table A2); the median age was 35(10) years. An age correction method, described above, was used to adjust HRV values of each control subject at the postoperative age of their matched IOE patient.

HRV parameters for each participant can be found in Appendix A Table A3.

### 3.2. Part 1: Evaluating the HRV in IOE Patients

#### 3.2.1. HRV Differences between IOE, TLE, and Healthy Control Groups

Table 4 shows the results of pre- and post-operative HRV values of IOE patients, TLE patients, and healthy controls. No statistically significant differences were found in all HRV parameters between the three groups for both studied periods.

Regarding the laterality of the epileptic focus, there was no significant difference for IOE and TLE groups.

There was also no difference between median pre- and post-operative HRV values within the IOE group as well as the TLE group.

#### 3.2.2. Correlation between HRV Parameters and the SUDEP-7 Score

We plotted the revised SUDEP-7 score against each HRV parameter for IOE and TLE groups. In the IOE group, we found a positive relationship between the revised SUDEP-7 score and pNN50 (r = 0.637, *p* = 0.008), and RMSSD (r = 0.579, *p* = 0.019). We then evaluated the effect of each factor of the SUDEP-7 inventory on HRV; we found that RMSSD and pNN50 values of patients who had at least one bilateral tonic-clonic seizure during the last year were significantly higher than those of patients without bilateral tonic-clonic seizure (*p* = 0.039 and 0.030 respectively). The difference between preoperative and postoperative HRV was not correlated with the presence of the bilateral tonic-clonic seizure.

As for TLE patients, we could not detect any relationship between HRV variables and SUDEP-7 scores. There was also no difference between HRV values and any individual factor of the SUDEP-7 inventory.

#### 3.2.3. Exploratory Subanalysis

##### IOE Group

**Figure 1:**
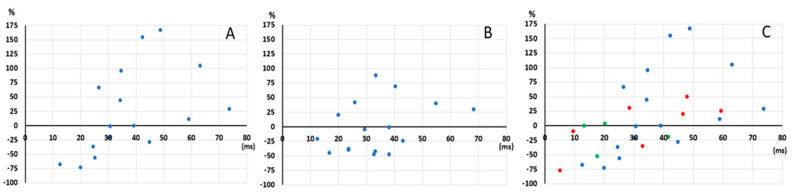
Comparison of pre-operative RMSSD values for each patient in the IOE group in the first part of the study (**A**), the TLE group (**B**), and the whole IOE group in the second part of the study (**C**) compared to its matched control. Each dot represents a patient. *x*-axis represent the pre-operative RMSSD of patients, *y*-axis represents the percentage of difference in RMSSD between patients (x) and their matched controls (c) [y = 100 ∗ (x − c)/c]. Insets A and B showed that the difference between IOE patients’ RMSSD from that of control seemed to be greater than in TLE patients. In inset C, blue dots represent IOE patients in the first part of the study, the green dots represent IOE patients with poor outcomes after surgery, and the red dots represent patients with temporo-insulo-opercular epilepsy. IOE = Insulo-opercular epilepsy; pNN50 = percentage of successive RR intervals that differ by more than 50 ms; RMSSD = root mean square of successive RR interval differences; SUDEP = sudden unexpected death in epilepsy.

Since RMSSD was considered the primary HRV measure in previous studies [47,48], it was examined in more detail for each patient. First, we plotted pre-operative RMSSD of IOE patients against the difference from the value of their matched controls (Figure 1A). We noted that the RMSSD value of IOE patients were either very high or very low compared to their matched controls. Eight patients had RMSSD values which differed by more than 50% from those of matched healthy control: five patients (31.25%) had higher values and three patients had lower values. Only three patients had an RMSSD value which differed by less than 25% from that of their matched controls.

**Figure 2:**
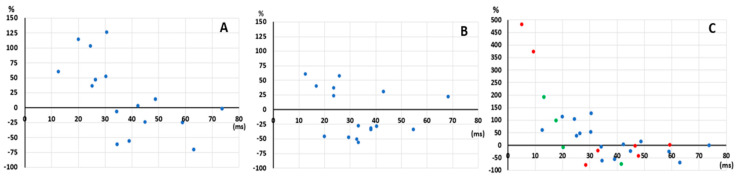
Comparison of pre-operative and post-operative RMSSD of each patient in the OIE group (**A**), TLE group (**B**), and IOE group in the second part of the study (**C**). Patients in each group were divided into two subgroups based on whether their pre-operative values were below or above the median RMSSD value (31 ms) of a population of 1315 healthy subjects aged 25 to 49 years (Voss et al., 2012). In the IOE group, low RMSSD tended to increase post-operatively, whereas high values tended to decrease after surgery. This pattern of HRV changes was not seen in the TLE group. (*x*-axis represents the pre-operative RMSSD measures; *y*-axis represents the percentage of difference between pre-operative (x) and post-operative RMSSD (p) out of pre-operative value (y = 100 ∗ (p − x)/x). Each dot represents a patient. In inset C, blue dots represent IOE patients in the first part of the study, the green dots represent IOE patients but poor seizure outcome after surgery, and the red dots represent patients with temporo-insulo-opercular epilepsy. IOE = Insulo-opercular epilepsy; TLE = Temporal lobe epilepsy; pNN50 = percentage of successive RR intervals that differ by more than 50 ms; RMSSD = root mean square of successive RR interval differences.

Next, we looked at the behavior of RMSSD pre- and post-operatively for each individuals (Figure 2A). Two different trends were observed: patients with a low pre-operative RMSSD value appeared to have an increase in RMSSD post-operatively, while patients with a high pre-operative RMSSD value appeared to have a decrease in RMSSD post-operatively. We used the median RMSSD value (30.81~31 ms) of the healthy population aged 25 to 49 years from the study of Voss et al. (2012) to divide the IOE group into two subgroups (Group 1a versus Group 1b) based on whether their preoperative RMSSD values were lower or higher than that threshold (Table 5). In group 1a (*n* = 7), RMSSD, and HF values were significantly lower than those of the control group preoperatively (*p* = 0.002; 0.007 and 0.018 respectively). On the other hand, all HRV parameter values were significantly higher for group 1b (*n* = 9) compared to the control group (*p* = 0.027; 0.024; 0.031 and 0.047). After surgery, all HRV values in group 1a significantly increased (*p* < 0.05) even though postoperative median values were not statistically different from those of matched controls. On the contrary, in group 1b, all HRV values decreased postoperatively, but only RMSSD and pNN50 met statistical significance. As for group 1a, all postoperative HRV values of group 1b were not statistically different from those of control subjects.

##### TLE Group

Using the same methodology for the TLE group, we noted that only two TLE patients (12.5%) had very high RMSSD values than their matched controls (Figure 2B). When examining the behavior of pre- and post-operative RMSSD, we also noted that some TLE patients had an increase while others had a decrease in RMSSD postoperatively. However, the difference between pre- and post-operative values appeared to be less than what was observed in the IOE group. We used the threshold of 31 ms to divide the TLE group into two subgroups (Group 2a versus Group 2b) and found no statistically significant changes pre- versus post-operatively in TLE patients with either low or high preoperative RMSSD values.

Although, in the TLE group there was no relationship between the SUDEP-7 score and the HRV parameters, the median SUDEP-7 score of group 2a (with RMSSD values < 31 ms) was significantly higher than that of group 2b (RMSSD value > 31 ms) (4 (2) vs. 1 (2); *p* = 0.014).

### 3.3. Part 2: HRV in All Patients with an Epileptogenic Zone Involving the Insulo-Opercular Region Irrespective of Post-Surgical Seizure Outcome

To assess HRV changes associated with insulo-opercular seizures on a larger set of patients, we included in this second part not only the 16 patients from part 1 with a definite IOE (as confirmed by a good surgical outcome followed a surgery limited to the insulo-opercular region) but also four more with a poor outcome (probable but not definite IOE—Appendix A Table A4), as well as 7 other patients who had a more extensive epileptogenic zone involving temporo-insulo-opercular structures (and undergoing temporo-insulo-opercular epilepsy surgeries, referred to as having IOE ‘plus’). This allowed us to assess HRV changes on a larger set of patients with insulo-opercular seizures, albeit more heterogenous than in part 1 of the study.

#### 3.3.1. Differences in HRV Values between Patients with an Epileptogenic Zone, which Included the Insulo-Opercular Region versus Healthy Controls

We performed the same statistical tests as in the first part and found no significant difference between HRV values of the group of patients with an epileptogenic zone, which included the insulo-opercular region (i.e., definite IOE, probable IOE and IOE ‘plus’ patients) with those of the control group both in the pre- and post-operative periods. Furthermore, within the former group, there was no significant difference between preoperative and postoperative HRV values.

Once again, we used the threshold of 31 ms to divide the epileptic group into two subgroups, and similar observations were found as in part 1, i.e., patients with low pre-operative HRV values, which were significantly increased, whereas those with high pre-operative HRVs were significantly decreased post-operatively.

More importantly, all post-operative HRV parameters were not significantly different from those of matched controls.

#### 3.3.2. Other Subgroup Analyses

We further analyzed pre- and post-operative HRV by taking into account the side of foci, the presence of an epileptogenic lesion, and whether a limited operculo-insular surgery or a larger temporo-insulo-opercular epilepsy surgery was performed, and found no statistical differences.

#### 3.3.3. Correlation between HRV Parameters and the Risk of SUDEP

Unlike when analysis was limited to the IOE group, when including all patients within the epileptogenic zone, which included the insulo-opercular region (definite IOE, probable IOE and IOE ‘plus’ patients), no correlation was found between HRV parameters and the revised SUDEP-7 score.

## 4. Discussion

In this study, we sought to look at HRV changes in patients with IOE/IOE ‘plus’ before and after epilepsy surgery, and compare them with patients with TLE as well as healthy controls. To our knowledge, no other study has done this before.

### 4.1. HRV in IOE

We did not find significant differences in median HRV values between patients with definite IOE versus TLE patients and healthy controls. The analysis for patients in which the insulo-opercular area was a part of their epileptogenic zone (definite or probable IOE and IOE ‘plus’ patients) showed similar findings. We did not find any differences in HRV related to the side of the focus either. This was somewhat unexpected, since the insula is known to be part of the central autonomic network [49,50]. While median group values did not differ from healthy controls, examination of individual results within the IOE group showed that some patients appeared to have a decrease in parasympathetic tone (low in RMSSD, pNN50, and HF), while others appeared to have an increase in both parasympathetic and sympathetic tones (high in all parameters). In our study, 31.25% of IOE patients had very high RMSSD, 18.75% had a very low value, and 50% had less than 50% difference from those of control. These changes can potentially be explained by the fact that the insular cortex plays a role in both sympathetic and parasympathetic control [7,49,50,51], and that resulting HRV changes could vary from patient to patient according to the side and site of subinsular epileptogenicity. Recently, Chouchou et al. [7] provided a functional mapping of the insula’s autonomic cardiac activity through the intracortical stimulation of 47 epileptic patients. Findings indicated symmetric parasympathetic and sympathetic responses between the right and left insula, which were in line with our findings. They also reported a posterior predominance of sympathetic control, whereas parasympathetic control seemed more anteriorly positioned within the insula, which was not replicated in our study (data not shown) due to the low number of subjects (7/16 patients had an anterior insulectomy, 3/16 patients had a posterior insulectomy, 6/16 had a radical insulectomy)).

### 4.2. Effect of Insulo-Opercular Epilepsy Surgery on HRV

In our study, median pre-operative HRV values did not significantly change after insulo-opercular surgery. At first glance, these results appear to contradict findings from [45,46]. In 2016, Lacuey et al. [45] compared HRV parameters obtained one year before an unsuccessful left posterior insular and peri-opercular resection versus those acquired four years after the surgery in a 33-year-old patient. They reported a significant decrease in the mean of normal-to-normal heartbeats (NNM) (13.75%), SDNN (21.14%), RMSSD (48.96%), and HF (21.35%), and an increase in LF (63.65%) and LF/HF ratio (307.95%). Because the patient eventually died (SUDEP), the authors suggested that autonomic dysfunction from the failed insular surgery might have put him at additional risk for SUDEP. One could argue that HRV changes after his insulo-opercular surgery were due to persistent seizures from an unresected focus, especially his frequently focal to bilateral tonic-clonic seizures and not the resection of the insula itself. Indeed, Mativo et al. (2010) showed that patients with bilateral tonic-clonic seizures (an important risk factor of SUDEP [52]) had decreased RMSSD, HF, and increased LF/HF ratio [24].

In 2019, the same group looked at HRV changes in patients undergoing temporal epilepsy surgery with variable resection/damage to the insula [46]. Authors found a significant decrease in RMSSD and CV when the temporal lobe surgery was combined with partial (<25%; *n* = 7) or extensive (≥25%; *n* = 1) insular removal compared to temporal surgeries with no (*n* = 4) or marginal (*n* = 9) insular damage. Once again, because almost half of the patients had persistent seizures (12 ILAE outcome class 1–2/21 patients), one cannot exclude the possibility that HRV changes were related to ongoing seizures rather than surgical damage to the insula. Since they did not include a healthy control group, and the duration between pre- and postsurgical recordings was very variable (from 9 to 168 months), it was unclear whether pre- and post-operative HRV values were in the normal ranges. In any case, patients in their series were hardly comparable to those in our series. In the former, most patients had temporal lobe epilepsy and surgeries systematically included the temporal lobe, but only variable portions of the insula (none, marginal, partial, and only one with extensive insular removal). In the latter, all had IOE with only seven whose resection extended to the temporal.

In an exploratory subanalysis, we observed a normalization of low or high preoperative HRV values in individuals operated in the insulo-opercular region (either when surgeries were limited to the insulo-opercular region or when they included the temporal lobe as well). Thus, it may be that the removal of the insula has a positive impact on autonomic function in the long run, at least if such a resection leads to a good post-operative seizure outcome (as was the case in 23/27 of our patients). Potential explanations for such a recovery include (a) arrest of seizure activity in the epileptogenic insula and areas of propagation involved in the central autonomic network; (b) gradual compensation by the intact contralateral insula; (c) reduction or withdrawal of antiepileptic drug after successful epilepsy surgery; (d) reduction in co-morbid anxiety and depressive disorders, which may impact HRV as well; (e) improvement in quality of life and a favorable psychosocial outcome [53,54,55].

### 4.3. HRV in TLE

In our study, median pre-operative HRV values in the TLE group did not significantly differ from those with IOE, as well as healthy controls. Fewer than a dozen studies have previously looked at interictal HRV in TLE, generating conflicting results. Similar to us, Varon et al. [56] failed to demonstrate significant changes in HRV. Some have observed a decrease in parasympathetic tone, while others have shown a decrease in both parasympathetic and sympathetic tones [33,57,58,59]. Suorsa et al. suggested that HRV is progressively reduced in refractory TLE but remains stable in well-controlled TLE patients [32].

### 4.4. Effect of TLE Surgery on HRV

In our study, no significant changes were noted between median pre-operative and post-operative HRV parameters. This is in line with findings from Persson et al. [60] who found that TLE surgery did not affect HRV. In the latter study, patients with a good surgical outcome had similar HRV values than control subjects while patients with poor outcome had lower values; one year after TLE surgery, post-operative HRV was not different from pre-operative HRV. Although the poorer outcome group had lower HRV post-operatively versus matched controls, this was already the case before the surgery. In contrast, Dericioglu et al. [33] found a reduction in total HRV after surgery with the sympathovagal balance altered towards the sympathetic side and Hilz et al. [34] found a reduction of sympathetic cardiovascular modulation after surgery. Possible reasons for such contradictory results include an inconsistent protocol for measurement and reporting of HRV, confounding factors (i.e., age, sex, medications, comorbid medical conditions), and the small numbers of patients, as discussed in a recent review by Myers et al. [47]

### 4.5. Correlation between Parasympathetic Parameters (RMSDD and pNN50) and the SUDEP-7 Score

In patients with definite IOE (first part of the study), we found a positive correlation between pNN50 and RMSSD with the SUDEP-7 score. This positive correlation between these parasympathetic parameters and the SUDEP-7 score may suggest that, in IOE, the increase in parasympathetic tone could be more worrisome than its decrease. This is partly supported by case reports of ictal bradycardia, atrioventricular block, or asystole in patients with insular epilepsy [4,61,62]. In the MORTEMUS study (Mortality in Epilepsy monitoring unit study), one near-SUDEP patient with insular epilepsy presented postictal cardiorespiratory arrest after a focal seizure and the other had ictal asystole [10]. On the other hand, in the TLE group, patients with low RMSSD values had significantly higher SUDEP-7 scores than those with high RMSSD values. This suggests that different types of focal epilepsies may have distinct effects on HRV parameters and could explain why previous studies have reported inconsistent results by lumping different forms of epilepsy (type, focus localization) [38,39,40]. Because the majority of patients included in our study had a good postoperative seizure outcome, it is unlikely that we will be able to assess in the long run whether those with either an increase or decrease in parasympathetic parameters have a higher risk of SUDEP. Further prospective work will be required to validate this observation.

When evaluating each factor of the SUDEP-7 inventory in the IOE group, we found that the influence of each factor on HRV parameters was different. While the presence of at least one bilateral tonic-clonic seizure was significantly associated with high values of two HRV parameters (pNN50 and RMSSD), the others did not affect any HRV parameters. These results were not replicated in the TLE group, possibly because of the low prevalence of bilateral tonic-clonic seizures in that group (4/16 versus 7/16~in the IOE group). This suggests that HRV may rather reflect the severity of the chronic cardiac consequence of generalized/bilateral tonic-clonic seizures, rather than the combination of many factors. In a case series of 25 drug-refractory epileptic patients, Novak et al. [38] reported two SUDEP cases who had low RMSSD with high and moderate SUDEP-7 scores (score of 7 and 4), both of whom had more than three generalized tonic-clonic seizures over the last year. Similarly, although Baysal-Kirac et al. [40] found no correlation between HRV parameters and the SUDEP-7 score in their series of 47 drug-resistant patients with focal/multifocal epilepsy, the only patient who died (definite SUDEP) did have a very low RMSSD and frequent generalized tonic-clonic seizures in the past year. If the factor mainly affecting HRV is represented by bilateral tonic-clonic seizures, then the control group in a future multicenter study might include patients with idiopathic generalized epilepsy and tonic-clonic seizures.

### 4.6. Limitations and Future Perspectives

We acknowledge that our study has several limitations, mainly related to its retrospective design: the need to resort to ECG recordings obtained during pre-operative VEEG sessions (rather than dedicated ECG recording sessions), variable time intervals between surgery and post-operative ECG acquisitions, and variable antiseizure medications between patients. We also recognize that the trend towards normalization observed in IOE subgroups could reflect regression to the median rather than a true effect from successful epilepsy surgery. However, this effect was only noted in the IOE group and not in the TLE group, which could support a real contribution from successful insulo-opercular epilepsy surgery. Even if the regression to the mean is genuinely responsible for these observed findings, the good news is that the insular resection does not appear to induce further HRV impairments in patients with IOE. We also recognize that in part 1 of our study, it would have probably been preferable to only include IOE patients with Engel 1 outcomes (leaving out the only patient with an Engel 2 outcome) for a more reliable comparison with TLE patients (who all Engel 1 outcomes); however, when this patient was excluded from the analysis, the results were unchanged (data not shown). We also acknowledge that our IOE and TLE groups, while matched for age and sex, were unmatched for lateralization of the epileptic focus. Finally, we are also aware of the implications of conducting an exploratory study with a modest number of patients in which several pairwise comparisons were conducted and for which an accurate correction to control for Type I errors could not be estimated.

Nevertheless, we deemed that some of the significant observations found with low *p*-values could be interesting enough to be worth reporting to encourage further work. On the other side, one must contend with the fact that OIE is relatively rare. To reach adequate power would require a multicenter collaboration. A standard protocol among sites for ECG recording and HRV analyses, both pre- and post-operatively, would need to be set in place to ensure that autonomic activity is assessed at a resting state, during sleep stages, and during autonomic activation tests. Adding other biomarkers for sudden cardiac death, such as corrected QT dispersion and T-wave alternans in refractory epilepsy, may improve the ability to identify the patients at high risk. Such better-designed studies should obviously not be limited to IOE, but should also include other types of epilepsy.

## 5. Conclusions

In refractory IOE, our preliminary findings suggest that HRV may be either lower in parasympathetic tone or higher in both parasympathetic and sympathetic tones. Higher parasympathetic tone was also positively correlated with the revised SUDEP-7 inventory score. More importantly, we found no evidence that insulo-opercular surgeries lead to major autonomic dysfunction when a good seizure outcome is reached. Hence, when one is convinced that the seizure focus lies in the insula and believes that a good outcome can be reasonably attained with surgery rather than medical therapy, epilepsy surgery in this region is justified. The same goes for refractory temporal lobe epilepsy, as temporal lobectomies leading to a good outcome do not negatively impact on HRV. Our findings may pave the way for a multicenter prospective study with a larger number of patients that could ultimately impact patient care in favor for earlier surgical intervention for eligible candidates and better long-term monitoring of cardiac markers of SUDEP risk.

## Figures and Tables

**Table 1:** Summary of presurgical, surgical and postsurgical data of IOE patients.

Pt	S/Lat	AO (yo)	Presurgical Period		D1 (Years)	Postsurgical Period		SUDEP-7 Score at A1	Presurgical Evaluation			Surgery	
			A1 (yo)	ASM		D2 (Months)	ASM		Operculo-INS Lesion on MRI	MEG	icEEG	Resected Areas	Engel
1	F/R	13	46	TPM, CBZ, LEV, LCM	33	64	LEV, LCM	6	yes	no spike	N/A	R superior INS + F-P opercula	Ia
2	F/L	10	19	OXC, PHT, PER	9	6	PHT, OXC	2	no	L operculo-INS	L ant-INS, F-operculum	L ant-INS	IId
3	M/L	29	37	PHT, CLB	8	142	None	2	no	L post-INS, L mesial T	L post-INS	post-INS	Ib
4	F/L	4	31	LEV, CBZ, CLB	27	29	CBZ, CLB	3	yes	no spike	N/A	L INS + F-P opercula	Ia
5	F/L	5	36	LCM, CLB, CBZ, TPM	31	25	CBZ	6	no	L F-T opercula-INS	L ant-INS, IFG, STG	L 2/3 ant-INS + partial F-T opercula	Ia
6	F/L	3	49	CBZ, LTG, CLB	46	12	CBZ, LTG, CLB	5	yes	L post-INS, P operculum	L post-INS	L INS	Ia
7	M/R	26	35	PHT, LTG	9	60	OXC	2	no	R F operculum, ant-INS	R F operculum (Note: ant INS electrode in wrong position	R ant-INS + F operculum	Ia
8	F/R	18	37	CBZ, LTG	19	204	CBZ, CLB	2	yes	NA	NA	R INS + F-P opercula	Ia
9	F/R	9	27	TPM, LCM	12	58	None	2	no	Junction between R ant-INS and orbitoF	Junction between R ant-INS and orbitoF	R ant-INS + F operculum	Ia
10	M/L	16	30	CBZ, CLB	14	28	CBZ	2	No (but subtle T2/FLAIR hypersignal in ant-INS, hypometabolic on PET)	L orbito-F	N/A	L lateral orbito-frontal cortex and operculum + ant-INS (pathology showed dyslamination)	Ia
11	M/L	24	33	LTG, CBZ	9	6	LTG, CBZ	2	yes (L ant-INS cavernoma)	N/A	N/A	L INS	Ia
12	F/R	5	24	TPM, CLB, LCM	19	70	OXC, TPM, CLB	3	yes (TSC)	R operculo-INS	N/A	R INS + F-P-T opercula	Ib
13	F/R	21	35	CBZ, TPM	14	18	CBZ, TPM	1	no	R superior ant-INS, F operculum, STG	R ant-INS, IFG	R ant-INS + F operculum	Ia
14	M/R	35	46	PGB, OXC	11	93	PGB, LCM, CNZ	2	no	cluster in R ant-INS/R orbitofrontal operculum; a few dipoles in medial frontal gyrus	Diffuse, non-localizing	R ant-INS + F operculum + orbitoF	Ia
15	F/R	14	32	LTG, CBZ, CLB	18	66	CBZ, LTG	4	no	R inf pre-CG, R mesial T	R inf CG, post INS	R inf central gyrus, R post INS	Ia
16	F/R	10	21	OXC, TPM, LTG, CLB	11	4	BRV	3	yes	NA	R T operculum, inf + ant INS	R T + F opercula +INS	Ia

Pt = patient, S = sex, Lat = lateralization of the epileptic focus, AO = age of onset, A1 = age at preoperative period, yo = years old, D1 = epilepsy duration; D2 = Elapsed time from surgery to postsurgical ECG; TCS = tonic-clonic seizures, ASM = antiseizure medication; Epilepsy duration = from onset to preoperative EEG; MRI = magnetic resonance imaging, PET = positron emission tomography, iSPECT = ictal single photon emission computed tomography, MEG = magnetoencephalography, icEEG = intracranial electroencephalography, M = male; F = female; R = right, L = left, INS = insula, F = frontal, P = parietal, T = temporal, CG = central gyrus, STG = superior temporal gyrus, IFG = inferior frontal gyrus, MFG = middle frontal gyrus, ant = anterior; post = posterior; inf = inferior; LEV = Levetiracetam, LTG = Lamotrigine, TPM = Topiramate, CBZ = Carbamazepine, OXC = Oxcabazepine, LCM = Lacosamide, PER = Perampanel, PHT = Phenytoin, CLB = Clobazam, CNZ = Clonazepam, PGB = Pregabalin, NA = not available.

**Table 2:** Summary of presurgical and postsurgical features of TLE patients.

Pt	S	Lat	AO (yo)	D1 (Years)	Presurgical Period		Postsurgical Period		SUDEP-7 Score at A1
					A1 (yo)	ASM	D2 (Months)	ASM	
T1	F	L	33	9	42	LTG, CLB, VPA, PGB	92	NONE	2
T2	F	L	2	19	21	CBZ, LEV	14	NONE	1
T3	M	L	19	15	34	LEV, LTG, CLB	41	LEV, LTG	3
T4	F	L	27	4	31	Pb, PGB, PHT	104	PGB, LCM	2
T5	F	R	2	32	34	PHT, TPM, LTG	77	PHT, LCM	5
T6	F	L	0.7	39	40	VPA, CBZ	120	NONE	4
T7	M	L	11	22	33	TPM, LTG	22	LTG, CLB	1
T8	F	L	13	26	39	CBZ	13	CBZ	1
T9	F	R	25	4	29	LCM, LEV, OXC	16	LEV, OXC	2
T10	M	R	25	3	28	LTG, LCM, LEV, LZP	64	LCM, CLB	4
T11	M	R	17	15	32	CBZ	24	CBZ, LZP	1
T12	F	L	23	1	24	LTG, LEV, CLB	60	LTG	2
T13	F	L	33	3	36	CLB, TPM, OXC, LTG	48	OXC, TPM, CLB	4
T14	M	L	30	16	46	OXC, BRV, Pb	71	OXC, Pb, GPN, LCM	4
T15	F	L	4	24	28	TPM, LTG	79	LEV	1
T16	F	R	19	3	22	TPM	18	OXC	3

Pt = patient, S = sex, Lat = lateralization of the epileptic focus, AO = age of onset, A1 = age at preoperative period, yo = years old, D1 = epilepsy duration; D2 = Elapsed time from surgery to postsurgical ECG TCS = tonic-clonic seizures, ASM = antiseizure medication, VPA = Valproic acid, LEV = Levetiracetam, LTG = Lamotrigine, TPM = Topiramate, CBZ = Carbamazepine, OXC = Oxcabazepine, LCM = Lacosamide, PER = Perampanel, PHT = Phenytoin, CLB = Clobazam, CNZ = Clonazepam; GPN = Gabapentin, PGB = Pregabalin; Pb = Phenobarbital; SUDEP = sudden unexpected death in epilepsy.

**Table 3:** Demographic and clinical features of patients whose epileptogenic zones involved the insulo-opercular area (*n* = 27).

**Preoperative age (years)** (Median; IQR; range)	35 (10) range (19–54)
**Postoperative age (years)** (Median; IQR; range)	38 (15) range (19–56)
**Gender (Number)**	
Male	9
Female	18
**Lateralization of** **epileptic focus (Number)**	
Right	15
Left	12
**Epilepsy duration (years)** **Median (IQR)**	range (6–42)
**Revised SUDEP-7 score** **median (IQR)**	2 (2) range (1–6)
**Elapsed time from** **surgery to postoperative EKG (months)**	29 (58) range (4–204)
**Resected zone (Number)**	
Limited to insulo-opercular areas	20
Temporo-insulo-opercular areas	7
**Surgical outcome (Number)**	
Good outcome	23
Poor outcome	4

Abbreviation: IQR = interquartile range.

**Table 4:** Pre- and post-operative HRV values for IOE, TLE and healthy control groups.

Studied Periods		Pre-Operative Period				Post-Operative Period			
Group	HRV Measures	RMSSD (ms)	pNN50 (%)	LF (ms^2^)	HF (ms^2^)	RMSSD (ms)	pNN50 (%)	LF (ms^2^)	HF (ms^2^)
IOE (*n* = 16)	Median	34.45	10.35	704.75	336.00	40.80	12.55	960.45	516.75
	IQR	22.30	23.18	489.13	651.68	25.80	18.65	1113.10	793.43
TLE (*n* = 16)	Median	32.95	9.4	851.55	325.40	25.50	4.45	507.50	194.80
	IQR	16.15	14.08	1144.00	400.77	17.80	11.30	730.35	204.90
CTL (*n* = 16)	Median	38.60	8.45	490.45	335.90	29.05	5.2	403.60	301.10
	IQR	36.45	25.20	709.08	859.35	33.50	20.50	754.75	404.05
*p* value (IOE vs. TLE vs. CTL)		0.625	0.776	0.538	0.646	0.246	0.231	0.124	0.163
Preop vs. postop	IOE group	0.679	0.796	0.121	0.918				
	TLE group	0.215	0.313	0.070	0.379				
**IOE group (*n* = 16)**									
**Group**	**HRV** **measures**	**Pre-Operative period**				**Post-Operative period**			
		**RMSSD** **(ms)**	**pNN50** **(%)**	**LF (ms^2^)**	**HF (ms^2^)**	**RMSSD** **(ms)**	**pNN50** **(%)**	**LF (ms^2^)**	**HF (ms^2^)**
L IOE (*n* = 7)	Median	34.60	7.80	691.40	424.00	42.80	9.50	979.00	1446.90
	IQR	18.40	23.80	502.30	600.90	36.70	25.20	1446.90	495.90
R IOE (*n* = 9)	Median	34.30	12.90	718.10	347.10	38.80	15.60	941.90	537.60
	IQR	25.80	22.55	1437.50	763.55	20.85	16.55	1015.15	590.50
CTL for L IOE (*n* = 7)	Median	31.00	7.30	565.10	326.80	22.70	2.60	314.00	156.90
	IQR	44.60	35.30	552.80	1754.40	35.00	28.70	907.70	821.40
CTL for R IOE (*n* = 9)	Median	39.10	10.70	270.90	388.90	32.00	5.50	461.10	326.80
	IQR	34.85	23.25	1168.40	791.30	35.75	17.45	516.75	422.90
*p* value	L vs. R	0.634	0.634	0.874	0.874	0.958	0.832	0.874	0.711
	L IOE vs. CTL	0.848	0.749	0.749	0.848	0.482	0.522	0.406	0.848
	R IOE vs. CTL	1.000	0.724	0.402	0.895	0.508	0.354	0.102	0.161
*p* value preop vs. postop	L IOE	0.866	1.000	0.176	0.866				
	R IOE	0.767	0.678	0.314	0.953				
**TLE group (*n* = 16)**									
**Group**	**HRV** **measures**	**Pre-Operative period**				**Post-Operative period**			
		**RMSSD** **(ms)**	**pNN50** **(%)**	**LF (ms^2^)**	**HF (ms^2^)**	**RMSSD** **(ms)**	**pNN50** **(%)**	**LF (ms^2^)**	**HF (ms^2^)**
L TLE (*n* = 12)	Median	29.55	7.35	632.15	237.15	26.90	4.80	507.50	194.80
	IQR	18.88	14.95	1140.70	447.45	13.93	10.38	730.35	190.87
R TLE (*n* = 4)	Median	35.40	12.75	1150.70	394.40	21.00	2.40	580.25	189.75
	IQR	11.55	11.33	1822.13	480.55	32.83	24.07	2704.60	1429.28
CTL for L TLE (*n* = 12)	Median	34.70	7.40	490.45	208.85	24.40	3.75	308.50	216.15
	IQR	31.63	16.70	639.95	802.98	40.53	21.08	771.73	424.60
CTL for R TLE (*n* = 4)	Median	48.00	18.85	577.50	565.15	35.20	7.55	580.40	401.85
	IQR	28.68	30.60	1715.75	1636.13	26.57	22.03	586.80	635.57
*p* value	L vs. R	0.467	0.467	0.275	0.225	0.72	0.67	0.90	1.00
	L TLE vs. CTL	0.773	0.862	0.817	0.773	0.861	0.795	0.356	0.862
	R TLE vs. CTL	0.917	0.602	0.175	0.602	0.386	0.386	1.000	0.386
*p* value preop vs. postop	L TLE	0.433	0.480	0.239	0.480				
	R TLE	0.73	0.715	0.144	0.715				

IOE = insulo-opercular epilepsy; TLE = temporal lobe epilepsy; CTL = control; R = right; L = left; IQR = interquartile range.

**Table 5:** Preoperative and postoperative HRV values of IOE subgroups compared to those of matched controls.

Group	HRV Measures	Preoperative Period				Postoperative Period			
		RMSSD (ms)	pNN50 (%)	LF (ms^2^)	HF (ms^2^)	RMSSD (ms)	pNN50 (%)	LF (ms^2^)	HF (ms^2^)
Group 1a (*n* = 7)	Median	25.20	2.40	281.80	248.70	42.80	15.60	1378.80	537.60
	IQR	10.40	7.50	538.30	157.80	15.40	17.60	1135.60	402.10
CTL 1a (*n* = 7)	Median	39.20	12.30	590.30	488.50	38.40	10.80	565.10	343.80
	IQR	19.20	23.40	2037.10	861.90	41.00	23.80	708.70	390.30
*p* value: Group 1a vs. CTL 1a		0.002	0.007	0.142	0.018	0.749	0.749	0.142	0.406
*p* value: Preop vs. postop		0.018	0.018	0.018	0.043				
Group 1b (*n* = 9)	Median	44.90	20.80	763.70	797.70	34.00	9.50	801.20	495.90
	IQR	24.20	19.95	1093.00	933.95	31.80	23.40	1372.90	1077.55
CTL 1b (*n* = 9)	Median	23.80	1.20	314.00	122.20	19.60	1.50	314.00	122.20
	IQR	28.85	19.00	483.80	595.65	27.75	16.95	612.55	550.20
*p* value: Group 1b vs. CTL 1b		0.027	0.024	0.031	0.047	0.222	0.144	0.102	0.310
*p* value: Preop vs. postop		0.038	0.011	0.859	0.086				

Group 1a and Group 1b consisted of IOE patients whose preoperative RMSSD values were lower or higher than 31 ms respectively. CTL = control; IQR = interquartile range; Preop = preoperative; Postop = postoperative.

## Data Availability

The data presented in this study are available on request from the corresponding author. The data are not publicly available due to privacy restrictions.

## Appendix A

**Table A1:** The revised SUDEP-7 inventory scores of IOE and TLE patients.

SUDEP-7 Risk Factor Inventory (Over Past 12 Months) at Presurgical Period								
IOE Patient	More than 3TCS	1 or More TCS	1 or More sz of Any Type	>50 sz of Any Type/Month	Duration > 30 yrs	Current Use of 3 or More AEDs	Mental Retardation, IQ < 70, or too Impaired to Test	Score
I1	0	0	0	2	3	1	0	6
I2	0	0	1	0	0	1	0	2
I3	0	0	0	2	0	0	0	2
I4	0	1	1	0	0	1	0	3
I5	0	1	1	0	3	1	0	6
I6	0	0	1	0	3	1	0	5
I7	0	0	0	2	0	0	0	2
I8	0	0	0	2	0	0	0	2
I9	0	0	0	2	0	0	0	2
I10	0	1	1	0	0	0	0	2
I11	0	0	0	2	0	0	0	2
I12	0	1	1	0	0	1	0	3
I13	0	0	1	0	0	0	0	1
I14	0	1	1	0	0	0	0	2
I15	0	1	0	2	0	1	0	4
I16	0	1	1	0	0	1	0	3
**TLE patient**	**More than 3TCS**	**1 or more TCS**	**1 or more sz of any type**	**>50 sz of any type/month**	**Duration > 30 yrs**	**Current use of 3 or more AEDs**	**Mental retardation, IQ < 70, or too impaired to test**	**Score**
T1	0	0	1	0	3	0	0	4
T2	0	0	1	0	3	1	0	5
T3	0	0	1	0	0	0	0	1
T4	0	0	1	0	0	1	0	2
T5	0	0	1	0	3	1	0	5
T6	0	0	1	0	3	1	0	4
T7	0	0	1	0	0	0	0	1
T8	0	0	1	0	0	0	0	1
T9	0	0	1	0	0	1	0	2
T10	2	0	1	0	0	1	0	4
T11	0	0	1	0	0	0	0	2
T12	0	0	1	0	0	1	0	2
T13	2	0	1	0	0	1	0	4
T14	2	0	1	0	0	1	0	4
T15	0	0	1	0	0	0	1	1
T16	20	0	1	0	0	0	0	3

SUDEP = sudden unexpected death of epilepsy; IOE = insulo-opercular epilepsy; TLE = temporal lobe epilepsy; TCS = tonic-clonic seizure; sz = seizure; yrs = years; AED = antiepileptic drugs; IQ = intelligence quotient.

**Table A2:** Age-, sex matched controls for patients’ groups at pre- and post-operative periods.

First Part										
IOE Group				Pre-op Matched Control		Post-op Matched Control		Matched TLE Group		
Code	S	Preop Age	Postop Age	Code	Age	Code	Age	Code	Preop Age	Postop Age
I1	F	47	54	C25	49	C1	53	T1	43	50
I2	F	19	19	C26	19	C26	19	T2	21	22
I3	M	38	49	C30	37	C24	44	T3	35	41
I4	F	32	36	C14	31	C9	36	T4	31	32
I5	F	36	40	C21	36	C2	39	T5	43	50
I6	F	38	50	C2	38	C5	52	T6	40	50
I7	M	36	42	C23	35	C8	40	T7	33	40
I8	F	37	54	C15	38	C7	52	T8	39	42
I9	F	28	33	C28	27	C14	31	T9	29	30
I10	M	31	35	C4	33	C27	33	T10	28	35
I11	M	34	35	C20	33	C29	38	T11	32	36
I12	F	23	28	C6	24	C18	28	T12	24	29
I13	F	35	37	C33	36	C22	36	T13	38	41
I14	M	46	54	C24	44	C10	53	T13	46	50
I15	F	32	38	C31	32	C15	38	T15	28	36
I16	F	21	23	C3	20	C19	23	T16	22	26
**Second Part**										
**IOE Group**				**Preop Matched Control**						
**Code**	**S**	**Preop Age**	**Postop Age**	**Code**	**Age**					
I1	F	47	54	C25	49					
I2	F	19	19	C26	19					
I3	M	38	49	C30	37					
I4	F	32	36	C14	31					
I5	F	36	40	C21	36					
I6	F	38	50	C2	38					
I7	M	36	42	C23	35					
I8	F	37	54	C15	38					
I9	F	28	33	C28	27					
I10	M	31	35	C4	33					
I11	M	34	35	C20	33					
I12	F	23	28	C6	24					
I13	F	35	37	C33	36					
I14	M	46	54	C24	44					
I15	F	32	38	C31	32					
I16	F	21	23	C3	20					
I17	2	37	40	C9	36					
I18	2	39	46	C32	42					
I19	1	35	42	C27	33					
I20	2	54	56	C1	53					
I21	2	27	37	C28	27					
I22	2	52	54	C5	52					
I23	1	25	34	C11	27					
I24	2	30	36	C22	36					
I25	1	30	32	C17	29					
I26	2	23	38	C19	23					
I27	1	40	42	C29	38					

**Table A3:** HRV measures of each participant.

IOE Group	Pre-Operative HRV Measures				Post-Operative HRV Measures			
Subject	RMSSD (ms)	pNN50 (%)	LF (ms^2^)	HF (ms^2^)	RMSSD (ms)	pNN50 (%)	LF (ms^2^)	HF (ms^2^)
I1	39.10	18.00	314.10	144.70	17.10	.30	582.00	104.70
I2	20.00	.50	179.80	248.70	42.80	5.90	1529.70	193.20
I3	30.60	6.70	447.30	424.00	69.30	28.20	979.00	1305.20
I4	30.40	7.80	281.80	189.30	46.30	22.60	1780.10	595.30
I5	63.10	44.00	691.40	1396.80	18.90	3.00	95.90	32.50
I6	48.80	30.50	755.00	797.70	55.60	31.00	801.20	1292.80
I7	34.30	12.90	578.20	880.20	31.90	8.50	1263.90	680.70
I8	25.20	1.40	222.20	241.20	34.40	5.00	455.20	555.10
I9	24.50	2.40	746.60	347.10	49.80	20.80	1378.80	440.10
I10	44.90	20.10	808.00	526.40	34.00	9.50	2299.20	495.90
I11	34.60	7.20	784.10	196.80	13.20	.30	333.20	33.10
I12	59.00	28.10	2749.50	1032.80	44.00	19.10	941.90	1000.10
I13	12.60	.30	68.60	77.40	20.20	1.80	287.20	80.00
I14	42.30	20.80	763.70	364.90	43.40	16.40	484.10	395.10
I15	26.50	5.20	718.10	302.40	38.80	15.60	1590.80	537.60
I16	73.70	40.30	2647.60	3411.30	72.40	33.40	2926.30	2333.00
I17	17.70	.00	118.80	146.70	35.20	11.90	517.30	487.80
I18	13.20	.90	207.50	36.50	38.50	18.10	472.80	226.10
I19	41.60	21.60	2285.30	831.40	10.60	.20	110.00	32.60
I20	20.30	.00	38.80	208.80	18.60	.00	386.10	105.50
I21	46.60	27.40	559.10	1268.50	45.00	21.10	2592.20	749.60
I22	9.50	.00	297.00	20.10	44.90	15.20	799.80	846.70
I23	28.50	6.40	1260.80	214.40	6.00	.00	173.40	8.40
I24	59.40	29.80	1378.90	1462.00	60.20	35.30	866.70	1088.30
I25	33.00	12.50	503.60	408.00	25.80	4.60	785.20	246.40
I26	47.90	16.30	408.90	1114.00	27.70	3.70	337.80	311.30
I27	5.10	.00	31.80	4.30	29.70	6.80	372.50	315.30
**TLE Group**	**Pre-Operative HRV Measures**				**Post-Operative HRV Measures**			
**Subject**	**RMSSD (ms)**	**pNN50 (%)**	**LF (ms^2^)**	**HF (ms^2^)**	**RMSSD (ms)**	**pNN50 (%)**	**LF (ms^2^)**	**HF (ms^2^)**
T1	23.60	3.90	133.80	130.40	32.30	9.40	327.10	211.10
T2	38.20	16.50	374.50	559.80	25.10	4.40	504.40	163.70
T3	16.90	.50	397.20	78.90	23.70	1.40	510.60	178.50
T4	23.70	1.40	365.90	162.60	29.20	3.00	248.00	228.20
T5	29.40	6.00	836.00	430.30	15.50	.30	680.70	103.50
T6	25.90	5.20	1527.60	200.30	40.70	12.80	1143.00	283.20
T7	40.30	16.40	969.20	697.70	28.70	5.90	512.50	293.30
T8	33.20	9.50	1042.80	333.50	14.50	.20	496.30	74.70
T9	38.10	16.20	1465.40	358.50	25.90	4.50	479.80	276.00
T10	32.70	9.30	664.80	317.30	16.10	.20	202.70	48.50
T11	33.30	10.10	1740.10	274.00	24.00	5.20	1499.50	159.60
T12	68.40	35.00	867.10	477.50	83.60	49.70	1049.80	951.00
T14	20.00	1.90	232.30	69.20	10.80	.00	124.10	26.40
T13	54.80	31.50	1717.40	1024.20	35.90	12.70	960.70	373.80
T15	12.60	.30	68.60	77.40	20.20	1.80	287.20	80.00
T16	43.00	18.80	2884.50	934.10	56.00	30.90	2907.30	1896.70
**Control Group**	**HRV Parameters**							
**Subject**	**RMSSD (ms)**		**pNN50 (%)**		**LF (ms^2^)**		**HF (ms^2^)**	
C1	19.60		1.50		195.70		76.00	
C2	18.30		1.00		314.00		122.20	
C3	57.20		30.40		2308.00		741.40	
C4	62.90		40.10		866.80		2409.00	
C5	10.50		.00		178.50		51.00	
C6	52.90		28.60		1772.00		1049.50	
C7	13.80		.00		263.50		28.00	
C8	26.10		4.90		461.10		275.40	
C9	37.30		10.80		953.10		343.80	
C10	11.40		.00		346.10		43.60	
C11	21.90		1.50		336.20		136.00	
C12	40.70		21.90		658.50		627.50	
C131	12.30		.00		420.10		57.70	
C14	38.40		9.60		565.10		326.80	
C15	57.60		12.30		270.90		488.50	
C16	41.40		19.30		932.30		451.90	
C17	50.50		26.10		701.50		1000.70	
C18	59.20		31.10		1486.50		1955.40	
C19	32.00		5.50		595.70		476.90	
C20	17.70		1.20		523.40		105.00	
C21	30.80		7.30		288.20		345.00	
C22	47.30		24.10		972.20		461.90	
C23	23.80		.60		226.90		160.20	
C24	16.60		.30		195.70		98.20	
C25	39.10		10.70		175.50		110.10	
C26	72.80		36.50		3018.80		1876.60	
C27	51.60		29.00		1086.20		919.60	
C28	38.80		7.00		213.20		388.90	
C29	22.70		2.60		143.70		156.90	
C30	31.00		5.20		590.30		257.50	
C31	15.90		.70		457.50		80.40	
C32	13.20		.00		169.70		52.30	
C33	39.20		19.20		973.70		1188.70	

**Table A4:** Summary of presurgical, surgical and postsurgical data of four probable IOE patients.

Pt	S/Lat	AO (yo)	Presurgical Period		D1 (Years)	Postsurgical Period		SUDEP-7 Score at A1	Presurgical Evaluation			Surgery	
			A1 (yo)	ASM		D2 (Months)	ASM		Operculo-INS Lesion on MRI	MEG	icEEG	Resected Areas	Engel
17	F/R	20	37	LEV, LTG	17	43	LEV, LTG. ESL	1	no	Non-localizing	R Mid-, post-INS, F-P-T opercula	R INS + F-P-T opercula	IIIa
18	F/L	32	39	PGB	7	83	PHT	1	no	L ant-T, mid-, post-lateral T	L INS, F-T opercula	L INS + T-opercula sparing Wernicke’s area	IIIa
19	M/L	21	35	LTG, VPA	14	62	CBZ, PHT	4	yes	L STG, ant-INS, IFG	L ant-INS, IFG	Ant-INS (IFG was not resected re language area)	IIIa
20	F/L	12	54	PHT, CBZ, LEV	42	5	LEVLCMLTG	3	no	N/A (Both PET and ictal SPECT showed a L INS focus)	L post-INS,	L post-INS	IIIa

Pt = patient, S = sex, Lat = lateralization of the epileptic focus, AO = age of onset, A1 = age at preoperative period, yo = years old, D1 = epilepsy duration; D2 = Elapsed time from surgery to postsurgical ECG; TCS = tonic-clonic seizures, ASM = antiseizure medication; Epilepsy duration = from onset to preoperative EEG; MRI = magnetic resonance imaging, PET = positron emission tomography, MEG = magnetoencephalography, icEEG = intracranial electroencephalography; M = male; F = female; R = right, L = left, INS = insula, F = frontal, P = parietal, T = temporal, CG = central gyrus, STG = superior temporal gyrus, IFG = inferior frontal gyrus, MFG = middle frontal gyrus, ant = anterior; post = posterior; inf = inferior; LEV = Levetiracetam, LTG = Lamotrigine, TPM = Topiramate, CBZ = Carbamazepine, OXC = Oxcabazepine, LCM = Lacosamide, PER = Perampanel, PHT = Phenytoin, CLB = Clobazam, CNZ = Clonazepam, PGB = Pregabalin, NA = not available.
